# Characterization of Non-hormone Expressing Endocrine Cells in Fetal and Infant Human Pancreas

**DOI:** 10.3389/fendo.2018.00791

**Published:** 2019-01-09

**Authors:** Abu Saleh Md Moin, Chiara Montemurro, Kylie Zeng, Megan Cory, Megan Nguyen, Shweta Kulkarni, Helga Fritsch, Juris J. Meier, Sangeeta Dhawan, Robert A. Rizza, Mark A. Atkinson, Alexandra E. Butler

**Affiliations:** ^1^Larry L. Hillblom Islet Research Center, University of California Los Angeles, David Geffen School of Medicine, Los Angeles, CA, United States; ^2^Diabetes Research Center, Qatar Biomedical Research Institute, Doha, Qatar; ^3^Department of Pathology, University of Florida, Gainesville, FL, United States; ^4^Institute of Pathology, Division of Clinical and Functional Anatomy, Medical University of Innsbruck, Tyrol, Austria; ^5^St. Josef Hospital of the Ruhr-University Bochum (RUB), Bochum, Germany; ^6^Diabetes and Metabolism Research Institute, City of Hope, Duarte, CA, United States; ^7^Division of Endocrinology, Diabetes, Metabolism, and Nutrition, Mayo Clinic College of Medicine, Rochester, MN, United States

**Keywords:** beta-cell, endocrine cells, development, maturation, transcription factor, replication

## Abstract

**Context:** Previously, we identified chromograninA positive hormone-negative (CPHN) cells in high frequency in human fetal and neonatal pancreas, likely representing nascent endocrine precursor cells. Here, we characterize the putative endocrine fate and replicative status of these newly formed cells.

**Objective:** To establish the replicative frequency and transcriptional identity of CPHN cells, extending our observation on CPHN cell frequency to a larger cohort of fetal and infant pancreas.

**Design, Setting, and Participants:** 8 fetal, 19 infant autopsy pancreata were evaluated for CPHN cell frequency; 12 fetal, 24 infant/child pancreata were evaluated for CPHN replication and identity.

**Results:** CPHN cell frequency decreased 84% (islets) and 42% (clusters) from fetal to infant life. Unlike the beta-cells at this stage, CPHN cells were rarely observed to replicate (0.2 ± 0.1 vs. 4.7 ± 1.0%, CPHN vs. islet hormone positive cell replication, *p* < 0.001), indicated by the lack of Ki67 expression in CPHN cells whether located in the islets or in small clusters, and with no detectable difference between fetal and infant groups. While the majority of CPHN cells express (in overall compartments of pancreas) the pan-endocrine transcription factor NKX2.2 and beta-cell specific NKX6.1 in comparable frequency in fetal and infant/child cases (81.9 ± 6.3 vs. 82.8 ± 3.8% NKX6.1^+^-CPHN cells of total CPHN cells, fetal vs. infant/child, *p* = 0.9; 88.0 ± 4.7 vs. 82.1 ± 5.3% NKX2.2^+^-CPHN cells of total CPHN cells, fetal vs. infant/child, *p* = 0.4), the frequency of clustered CPHN cells expressing NKX6.1 or NKX2.2 is lower in infant/child vs. fetal cases (1.2 ± 0.3 vs. 16.7 ± 4.7 clustered NKX6.1^+^-CPHN cells/mm^2^, infant/child vs. fetal, *p* < 0.01; 2.7 ± 1.0 vs. 16.0 ± 4.0 clustered NKX2.2^+^-CPHN cells/mm^2^, infant/child vs. fetal, *p* < 0.01).

**Conclusions:** The frequency of CPHN cells declines steeply from fetal to infant life, presumably as they differentiate to hormone-expressing cells. CPHN cells represent a non-replicative pool of endocrine precursor cells, a proportion of which are likely fated to become beta-cells.

***Precis***: CPHN cell frequency declines steeply from fetal to infant life, as they mature to hormone expression. CPHN cells represent a non-replicative pool of endocrine precursor cells, a proportion of which are likely fated to become beta-cells.

## Introduction

In humans, most beta cell growth and development occurs during gestation and early life (1–3). Beta-cells are first detected at 9 weeks gestation, with fractional beta-cell area increasing linearly throughout gestation accompanied by a readily detectable frequency of beta-cell replication (1). This implies a key role of replication in the prenatal expansion of beta-cells and in establishment of beta-cell mass. After birth, a high frequency of beta-cell replication in infancy further contributes to the expansion of beta-cell mass, but replication of beta-cells declines rapidly with increasing age and, after the age of 3–5 years, is negligible in most cases (2). By contrast, beta-cell apoptosis has been reported to be low during mid-gestation, rising during the perinatal period and falling again in infancy (3); although later studies are contradictory, detecting a higher frequency of apoptosis during gestation (1) and no perinatal rise in apoptosis in humans (2).

Recently, we reported a high frequency of chromograninA-positive hormone-negative (CPHN) cells in fetal human pancreas, whose presence falls rapidly after birth, and these cells detectable only at very low frequency in adult non-diabetic human pancreas (4). Further, we have demonstrated that these cells are more frequent in the setting of both type 1 and type 2 diabetes, perhaps indicating an attempt, albeit insufficient, at regeneration of beta-cells lost to disease (4–6).

It has been established that endocrine cells arise from pancreatic and duodenal homeobox (PDX)-1-positive precursors during gestation (7, 8), and that beta-cell replication then plays a major role in establishing the beta-cell complement (1, 9–13). Contributions from other mechanisms, such as neogenesis and transdifferentiation, have been proposed (9, 14, 15). Moreover, Sarkar et al. have suggested that replication of existing beta-cells is insufficient to account for the increase in beta-cell mass during human gestation and therefore that a significant proportion of endocrine expansion during fetal life must derive from replication of hormone-negative precursors (13).

CPHN cells may represent a pool of endocrine cells, largely expressing beta cell transcription factors and a proportion of which are likely fated to become beta-cells. While they are present in highest frequency during gestation, they only occur at low levels throughout life, and their numbers can increase in response to endocrine deficiency, as seen in diabetes (16–18). The purpose of this study was to quantify the frequency of CPHN cells in human fetal and infant pancreas, as well as to characterize this population in terms of their replicative capacity and markers of endocrine cell identity.

## Materials and Methods

### Design and Case Selection

This study was carried out with approval from the Board for Ethical Issues at the Medical University of Innsbruck, the Mayo Clinic Internal Review Board, the University of California Los Angeles Internal Review Board and the University of Florida Institutional Review Board.

#### Mayo Clinic [Supplementary Table 1, (19)]

Sections of pancreas from fetal (*n* = 4), and infant subjects with age range <1–12 weeks, (*n* = 19), subjects were obtained from the Mayo Clinic autopsy archives with IRB permission (IRB# 15-004992). Potential cases were identified by retrospective analysis of the Mayo Clinic autopsy database. Exclusion criteria included pancreatic tissue that had undergone autolysis or showed features of pancreatitis.

#### Medical University of Innsbruck [Supplementary Table 1, (19)]

Sections of fetal pancreas from 4 fetal subjects were obtained from the Institute of Pathology and the Division of Clinical and Functional Anatomy, Medical University of Innsbruck. They were obtained from miscarriage and legal abortions including parental consent and in compliance with the local governmental and institutional guidelines.

Ten of the neonatal subjects from Mayo Clinic and the four fetal subjects acquired from the Medical University of Innsbruck were included in a previous publication (4) (Fetal cases 1–4 and infant/child cases 1–10, as listed in Supplementary Table 1, (19)).

#### University of Florida, Gainesville, Network for Pancreatic Organ Donors With Diabetes (nPOD) Program [Supplementary Tables 2, 3, (19)]

For analysis of replication and transcription factors NKX2.2 and NKX6.1 in CPHN cells, pancreatic sections from a subset of the nPOD donors [8 fetal [for replication], 7 fetal [for NKX6.1 or NKX2.2], 16 infant/child [for replication] and 10 infant/child donors [for NKX6.1 or NKX2.2] were acquired] [Supplementary Table 2, (19)]. For analysis of ductal replication and quantification of hormone-expressing cells in ductal structures (ducts and pancreatic duct glands), sections were acquired from 9 fetal donors (32–40 weeks gestation) and 23 infant/child donors (0.08–5 years) [Supplementary Table 3 (19)].

### Pancreatic Tissue Processing and Staining

#### Immunofluorescence Staining for Chromogranin Positive Hormone-Negative [CPHN] Cells

Four Micrometer paraffin tissue sections from each subject were stained for chromograninA, insulin, glucagon, somatostatin, pancreatic polypeptide, and ghrelin. Standard immunohistochemistry protocols were used for fluorescent immuno-detection of various proteins in pancreatic sections, as previously described (5).

In brief, one pancreas section obtained from the tail of pancreas was analyzed for each subject. Briefly, slides were incubated at 4°C overnight with a cocktail of primary antibodies prepared in blocking solution (3% BSA in TBST) at the following dilutions: mouse anti-glucagon (1:1,000, Sigma-Aldrich G2654-.2ML; St. Louis, MO); guinea-pig anti-insulin (1:200, Abcam7842; Cambridge, MA), rat anti-somatostatin (1:300, EMD Millipore MAB354; Billerica, MA), goat anti-pancreatic polypeptide (1:3,000, Everest Biotech; Ramona, CA), rat anti-ghrelin (1:50, R&D Systems MAB8200; Minneapolis, MN). The primary antibodies were detected by a cocktail of appropriate secondary antibodies (Jackson ImmunoResearch, Westgrove, PA) conjugated to Cy3 (1:200, for ChrgA), FITC (1:200 each, to detect glucagon, somatostatin, pancreatic polypeptide, and ghrelin) or Cy5 (1:100, to detect insulin). Slides were viewed using a Leica DM6000 microscope (Leica Microsystems, Deerfield, IL) and images were acquired using the 20x objective (200x magnification) using a Hamamatsu Orca-ER camera (C4742-80-12AG, Indigo Scientific, Bridgewater, NJ) and Openlab software (Improvision, Lexington, MA).

### Morphometric Analysis

One section of the pancreas per subject was stained with appropriate primary and secondary antibodies. Fifty islets per subject were imaged at 20x magnification. An islet was defined as a grouping of four or more endocrine cells. A cluster was defined as a grouping of three or fewer chromogranin A positive cells. Islets were selected by starting at the top left corner of the pancreatic tissue section and working across the tissue from left to right and back again in a serpentine fashion, imaging all islets in this systematic excursion across the tissue section. Analysis was performed in a blinded fashion (ASMM, CM, and AEB), and all CPHN cells identified were confirmed by a second observer. The endocrine cells contained within each islet were manually counted and recorded as follows: (1) the number of cells staining for chromogranin A, (2) the number of cells staining for the endocrine hormone cocktail, and (3) the number of cells staining for insulin. Thus, cells staining for chromogranin A but not the other known pancreatic hormones (insulin, glucagon, somatostatin, pancreatic polypeptide, or ghrelin) were noted. At 200x magnification, using the Leica DM6000 with a Hamamatsu Orca-ER camera and a 0.7x C-mount, each field of view was calculated to be 0.292 mm^2^. Within the fields imaged to obtain the 50 islets per subject, all single endocrine cells and clusters of endocrine cells (two or three adjacent endocrine cells) were counted and recorded as outlined above.

#### Assessment of Replication and Endocrine Transcription Factors in ChromograninA Positive Hormone-Negative [CPHN] Cells

To determine replication and transcription factor expression in CPHN cells, we utilized our previously published protocol (5). Briefly, we developed and used a new immunohistochemical staining technique involving monovalent F(ab′)2 fragments to distinguish between the two mouse primary antibodies. Here is a brief description of the protocol. After antigen retrieval of the paraffin sections of pancreas of fetal or infant cases (by following the standard antigen retrieval procedure using citrate buffer), tissues were then blocked with blocking buffer (3% BSA, 0.2% Triton X-100) for 1 h at room temperature followed by incubation with the first primary antibodies prepared in antibody buffer (3% BSA in TBST) at the following dilutions (mouse anti Ki67 [1:50, M7240; DAKO], mouse anti-Nkx6.1 [1:300, F55A10; DSHB], mouse anti- Nkx2.2 [1:50, 74.5A5; DHSB]) in separate sections at 4°C overnight. First primary antibody was detected by Cy3-conjugated donkey antimouse IgG (1:100, 715-166-150; Jackson ImmunoResearch). Slides were then incubated sequentially with mouse serum (5% [vol/vol], 015-000-120; Jackson ImmunoResearch) and unconjugated F(ab′)2 fragment of donkey antimouse IgG (40 μg/mL, 715-007-003; Jackson ImmonoResearch) for 1 h at room temperature. After each incubation, the slides were washed with 1xTBST and 1x TBS (10 min each). After that, slides were incubated with second primary antibody (mouse anti-glucagon, 1:2,000, G2654-.2ML; Sigma-Aldrich) at 4°C overnight. Second primary antibody was detected with donkey antimouse Alexa 647 (1:100, 715-606-151; Jackson ImmunoResearch). Finally, slides were incubated at 4°C overnight with a cocktail of third primary antibodies prepared at the following dilutions: guinea pig anti-insulin (1:100, 7842; Abcam), rat anti-somatostatin (1:100, MAB354;EMDMillipore), goat anti-pancreatic polypeptide (1:3,000; Everest Biotech), rat anti-ghrelin (1:50, MAB8200, R&D Systems); and rabbit anti-chromogranin A (1: 200, NB120-15160; Novus Biologicals). The third primary antibodies were detected by a cocktail of secondary antibodies [F(ab′)2 fragments]; donkey antiguinea pig Alexa 647 (1:100, 706-606-148, for insulin; Jackson ImmunoResearch), donkey antirat Alexa 647 (1:100, 712-606-153, for ghrelin and somatostatin; Jackson ImmunoResearch), donkey antigoat Alexa 647 (1:100, 705-606-147 for pancreatic polypeptide; Jackson ImmunoResearch), and donkey antirabbit fluorescein isothiocyanate (1:100, 711-096-152 for chromogranin A; Jackson ImmunoResearch). Slides were counterstained to mark the nuclei using a mounting medium containing DAPI (Vectashield; Vector Labs) and viewed using a Leica DM6000 microscope (Leica Microsystems), and images were acquired using the _20 objective (200x magnification) using a Hamamatsu Orca-ER camera (C4742-80-12AG; Indigo Scientific) and Openlab software (Improvision).

Slides were viewed and imaged as described above. CPHN cells (located in islets, clusters and in single cells) that express NKx6.1, NKx2.2, or Ki67 were identified (by following the similar procedure of detecting CPHN cells in the different compartments of pancreas) and documented blindly by two independent researchers (ASMM and CM).

#### Immunohistochemical Staining of Ductal Structures for Replication and Endocrine Cell Subtypes

Adjacent sections of pancreas were stained for (1) Ki67, insulin and glucagon and (2) insulin, somatostatin and pancreatic polypeptide. Primary antibodies used were mouse anti-Ki67 (1:50; Dako M7240; Carpinteria, CA), guinea pig anti-insulin (1:1,000; Dako A0564), rabbit anti-somatostatin (1:1,000; Dako A056601-2), mouse anti-glucagon (1:1,000; Abcam ab10988, Cambridge, MA;), rabbit anti-pancreatic polypeptide (1:800; Abcam ab113694). Insulin was developed using EnVision™ G|2 System/AP, Rabbit/Mouse (Dako K5355Ki67); Ki67 and somatostatin were developed using EnVision Detection Systems Peroxidase/DAB, Rabbit/Mouse, HRP (Dako K406511); glucagon and pancreatic polypeptide were developed using ImmPRESS™ HRP Universal Antibody (Anti-Mouse IgG/Anti-Rabbit IgG, Peroxidase) Polymer Detection Kit (Vector Laboratories MP-7500, Burlingame, CA) with HIGHDEF blue IHC chromogen (HRP) for color development (Enzo Life Sciences ADI-950-151-0030, Farmingdale, NY). Slides were counterstained with hematoxylin (1:10; Dako S330930-2).

### Morphometric Analysis

#### CPHN Quantification

Fifty islets per subject were imaged at 20x magnification. An islet was defined as a grouping of four or more chromograninA positive cells. A cluster was defined as a grouping of three or fewer chromograninA positive cells. The endocrine cells contained within each islet were manually counted as previously described (5).

The mean number of endocrine cells counted within islets for the fetal group was 782 ± 50 cells per subject and for the infant group was 1,514 ± 151 cells per subject. The mean number of cells counted in clusters for the fetal group was 156 ± 32 cells per subject, and for the infant group was 81 ± 7 cells per subject. The mean number of chromograninA positive hormone-negative [CPHN] cells per individual identified in islets from the fetal subjects was 47.3 ± 11.4 cells per individual and from the infant group was 15.6 ± 1.9 cells per individual. The mean number of CPHN cells per individual identified in clusters from fetal subjects was 47 ± 11 and from infant subjects was 15 ± 2 cells per individual. At 200x magnification, using the Leica DM6000 with Hamamatsu Orca-ER camera and a 0.7x C-mount, each field of view was calculated to be 0.292 mm^2^. Within the fields imaged to obtain the fifty islets per subject, all clusters of endocrine cells (one, two, or three adjacent endocrine cells) were counted and recorded as outlined above.

#### Quantification of Replication and Expression of NKX6.1 and NKX2.2 in CPHN Cells

To investigate the potential endocrine cell lineage of CPHN cells in fetal and infant/children pancreas, we evaluated Ki67 as a replication marker (mouse anti-Ki67, 1:50; RRID:AB_2142367; catalog no. M7240; Agilent Technologies), NKX2.2 as a panendocrine transcription factor (mouse anti-NKX2.2, 1:50; RRID:AB_531794; catalog no. 74.5A5; DHSB), and NKX6.1 as a β-cell transcription factor (mouse anti-NKX6.1, 1:300;RRID:AB_532378; catalog no. F55A10; DSHB) CPHN cells were identified as described previously (20). To assess replication and the presence of the transcription factors NKX2.2 and NKX6.1 in CPHN cells, 20 fields were viewed with a Zeiss Axioskop 2 microscope (Carl Zeiss Microscopy, Thornwood, NY) and images acquired using an Axiocam MR3 camera and Axiovision 4.0 software (Carl Zeiss Microscopy, Thornwood, NY). Using this microscope, camera and software, each field of view has an area of 0.42 mm^2^. CPHN cells that express Ki67 or NKX6.1 or NKX2.2 were counted in three different compartments (within islets, as clustered cells or as single cells) of a pancreas section. Data in islets were expressed as number of Ki67 or NKX6.1 or NKX2.2 cells per islet sections and in case of clustered or single cells, data were expressed as number of cells per mm^2^ area.

#### Quantification of Replication and Hormone Expressing Cells in Ductal Structures

Slides stained by immunohistochemistry were digitally scanned using Aperio ScanScope (Aperio Technologies, Vista, CA) and analyzed using Aperio ImageScope version 12.1.0.5029. Sections were examined and quantified in a blinded manner. Ductal structures embedded in the mesenchyme and having a pancreatic duct gland (PDG) compartment were identified as interlobular ducts. PDG compartments were identified as invaginations stemming from the interlobular ducts. The total number of cells in interlobular ducts and surrounding PDG compartments were counted by the Aperio software. The number of Ki67, insulin, glucagon, somatostatin and pancreatic polypeptide cells found in interlobular ducts and PDGs were counted manually. The mean number of interlobular duct cells counted in the fetal sections was 684 ± 200 (range 56–1,598) and in the infant/child sections was 2,122 ± 533 (range 232–11,057). The mean number of PDG cells counted in the fetal sections was 385 ± 152 (range 5–1,350) and in the infant/child sections was 927 ± 240 (range 42–3,593).

### Statistical Analysis

Statistical analysis was performed using the Student's *t*-test, two-way ANOVA or non-linear correlation analysis (where appropriate) with GraphPad Prism 6.0 software (GraphPad Software, La Jolla, CA). Data in graphs and Supplementary Tables (19) are presented as means ± SEM. Findings were assumed statistically significant at *P* < 0.05.

## Results

### Pancreas Morphology During Development

During gestation, endocrine cells are present in high density but occur mostly in small clusters rather than in well-formed islets; a very high frequency of replication is present in both endocrine and exocrine compartments (1). After birth, the density of endocrine cells is decreased, largely as a consequence of growth of the exocrine pancreas, and the majority of endocrine cells are now found in well-recognizable islets. The frequency of replication, whilst still high in infancy, is lower than in the fetal tissue (2). During early childhood, the frequency of replication decreases in the exocrine pancreas, and becomes negligible in the endocrine compartment after the age of 3 years, in most cases. With age, the morphology of the pancreas matures, with well-formed islets and a lobular exocrine structure as in the adult pancreas (Supplementary Figures 1–3).

#### ChromograninA Positive Hormone-Negative (CPHN) Cells Are Abundant in Late Fetal Life but Decrease During Infancy

**Figure 1:**
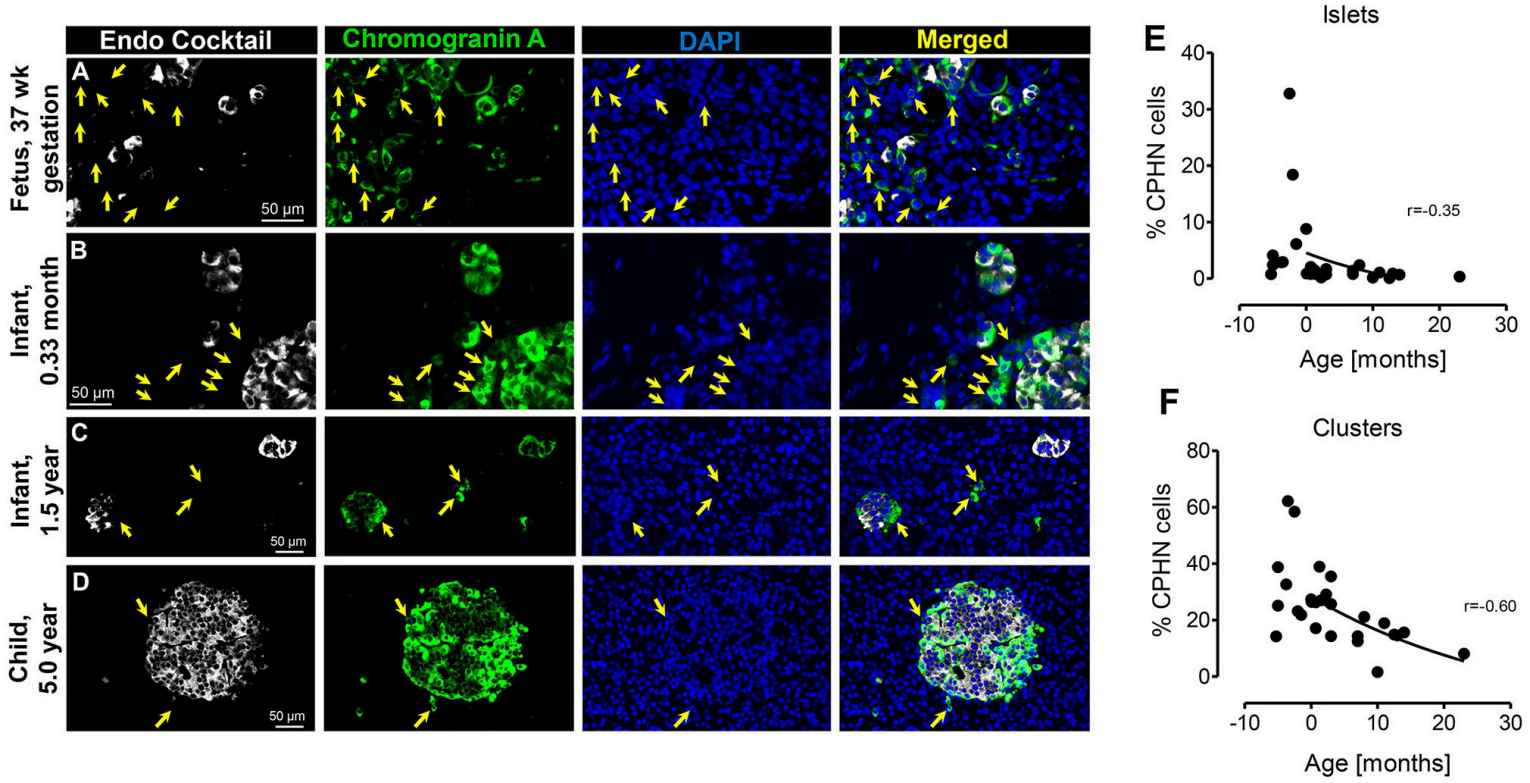
The frequency of chromograninA positive hormone-negative (CPHN) cells decreases with age. Representative pancreatic sections from fetal **(A)** and infant-child **(B–D)** cases immunostained for Endocrine cocktail (insulin, glucagon, somatostatin, pancreatic polypeptide, and ghrelin) (white), chromograninA (green), and DAPI (blue). Yellow arrows show CPHN cells. Frequency of CPHN cells in islets **(E)** and clusters **(F)**. Scale bars, 50 μm.

During gestation small clusters of pancreatic endocrine cells begin to assemble into islets that include a high proportion of CPHN cells (Figure 1A), confirming our earlier findings (4). This aggregation process of endocrine cells in the pancreas continues in the neonatal and early infancy period, where CPHN cells are still frequent (Figures 1B,C) and by 5.0 years of age the majority of endocrine cells in the pancreas are assembled into islets (Figure 1D) (2). For this analysis, cases already described in a previous publication (4) were also included here (Fetal cases 1–4 and infant cases 1–10) (19). CPHN cells were prevalent as single cells and in small clusters scattered throughout the exocrine pancreas in the fetal pancreas (Figure 1). However, the abundance of CPHN cells decreased during the transition from fetal to infant life both in islets (*r* = −0.35) (8.8 ± 3.9 vs. 1.4 ± 0.4%, fetal vs. infant, *p* < 0.01) (Figure 1E) and small clusters (*r* = −0.60) (34.5 ± 6.2 vs. 20.5 ± 2.2%, fetal vs. infant, *p* < 0.01) (Figure 1F). The steep decline in CPHN cells with age potentially indicates that CPHN complete the process of differentiation, becoming mature pancreatic endocrine cells during infancy.

#### CPHN Cells Do Not Replicate in Fetal and Infant Pancreas

**Figure 2:**
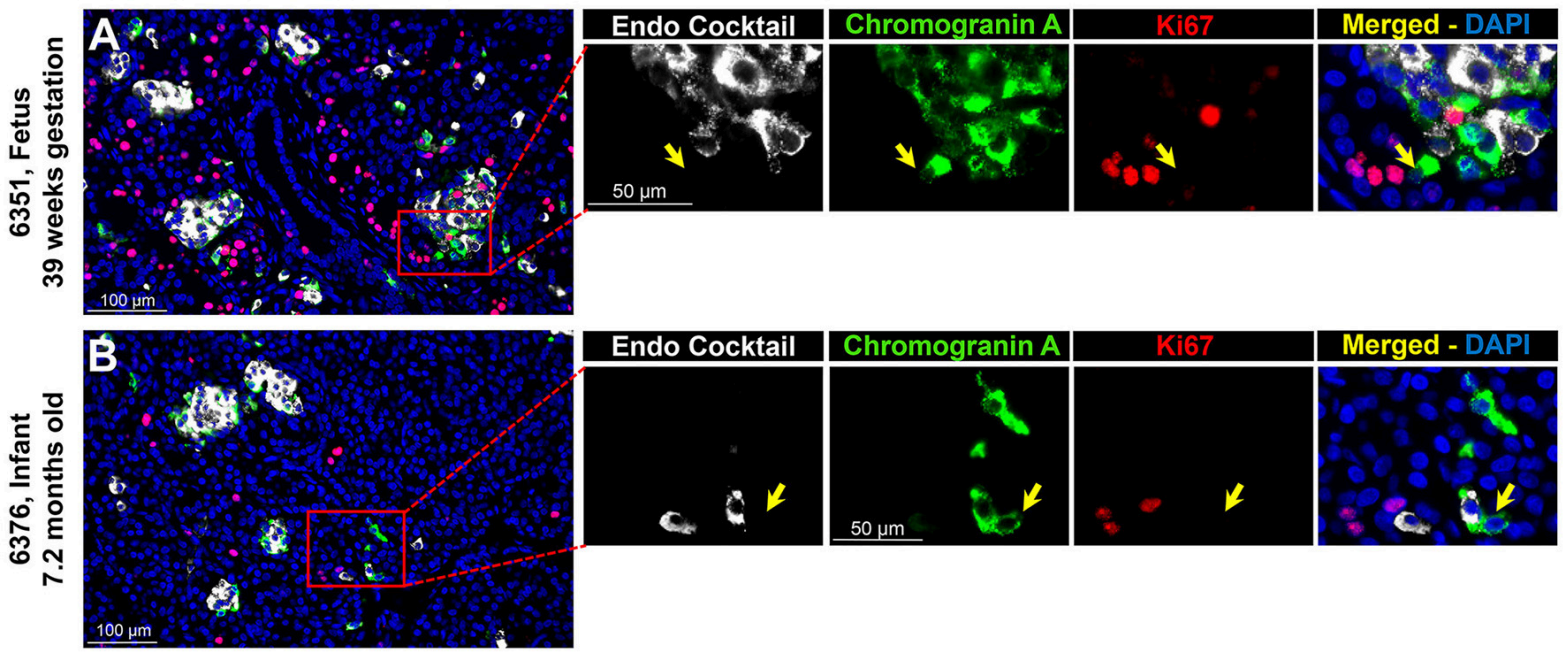
ChromograninA positive hormone-negative (CPHN) cells do not replicate during fetal and infant life. Representative pancreatic sections from fetal **(A)** and infant **(B)** donors immunostained for Endocrine cocktail (insulin, glucagon, somatostatin, pancreatic polypeptide, and ghrelin) (white), chromograninA (green), Ki67 (red), and DAPI (blue). Yellow arrows indicate CPHN cells. CPHN cells were rarely positive for Ki67 staining in both fetal and infant groups; no detectable difference was found in the frequency of replicative CPHN cells between fetal and infant pancreatic sections. Scale bars: 100 μm for low power and 50 μm for high magnification images.

Endocrine cells expand by replication during late fetal and early neonatal life in humans (2). Since CPHN cells may represent a precursor to fully differentiated endocrine cells, we quantified replication of CPHN cells in pancreatic tissue from fetuses and infants by use of Ki67, chromograninA and a cocktail of islet endocrine hormones (insulin, glucagon, somatostatin, pancreatic polypeptide, and ghrelin) (5). CPHN cells were rarely observed to replicate (0.2 ± 0.1 vs. 4.7 ± 1.0%, CPHN vs. islet hormone positive cell replication, *p* < 0.001), indicated by the lack of Ki67 expression in CPHN cells whether located in the islets or in small clusters, and with no detectable difference between fetal and infant groups (Figures 2A,B). Only in one fetal and one infant case were we able to find a CPHN cell expressing Ki67 [Supplementary Figures 4A,B, (19)].

The pancreatic duct gland (PDG) compartment within the pancreas is proposed to be a potential multipotent precursor compartment (21, 22), prompting us to examine replication, islet hormone positive cells, and CPHN cells within this compartment during development. The frequency of replication of the cells in interlobular ducts and PDGs was not different between the infant/child and fetal donor groups (Ducts: 5.06 ± 1.35 vs. 5.16 ± 1.33%, infant/child vs. fetus, *p* = ns; PDGs: 3.95 ± 0.81 vs. 2.38 ± 0.95%, infant/child vs. fetus, *p* = ns) (Supplementary Figures 5A,B). The percentage of cells that express insulin within these ducts was higher in the infant/child vs. the fetal donors in both the interlobular ducts (0.69 ± 0.12 vs. 0.31 ± 12%, infant/child vs. fetus, *p* < 0.05) and the PDGs (3.53 ± 0.92 vs. 0.58 ± 0.40%, infant/child vs. fetus, *p* < 0.01). No differences in frequency of glucagon, somatostatin, or PP-expressing cells were found in the interlobular duct compartment. In the PDG compartment, only PP expression differed, being more frequent in the fetuses (1.06 ± 0.32 vs. 3.63 ± 2.20%, infant/child vs. fetal, *p* < 0.05) (Supplementary Figures 5C,D).

CPHN cells were rare in the pancreatic ducts and pancreatic duct glands (PDGs) of the fetal and infant/child pancreas (0.15 ± 0.05% in ducts, 0.26 ± 0.09% in PDGs) and, when found, were not Ki67 positive [Supplementary Figures 6A,B, (19)], again emphasizing that this pool of hormone-negative endocrine cells does not replicate. In contrast, a proportion of cells (4.6 ± 0.9 vs. 1.2 ± 0.4, % of Ki67^+^ endocrine cells of total endocrine cells, fetal vs. infant/child, *p* < 0.01) [Supplementary Figure 7, (19)] expressing islet hormones (stained for the endocrine cocktail) were Ki67 positive (Supplementary Figures 8A,B, 19), and the percentage of these Ki67 positive endocrine cells decreased from fetal to postnatal life [Supplementary Figure 8C, (19)], in accord with the frequency of beta-cell replication reported by Kassem et al. (3).

#### Islet CPHN Cells Display the Transcriptional Program for Islet Endocrine Identity During Pancreatic Development

**Figure 3:**
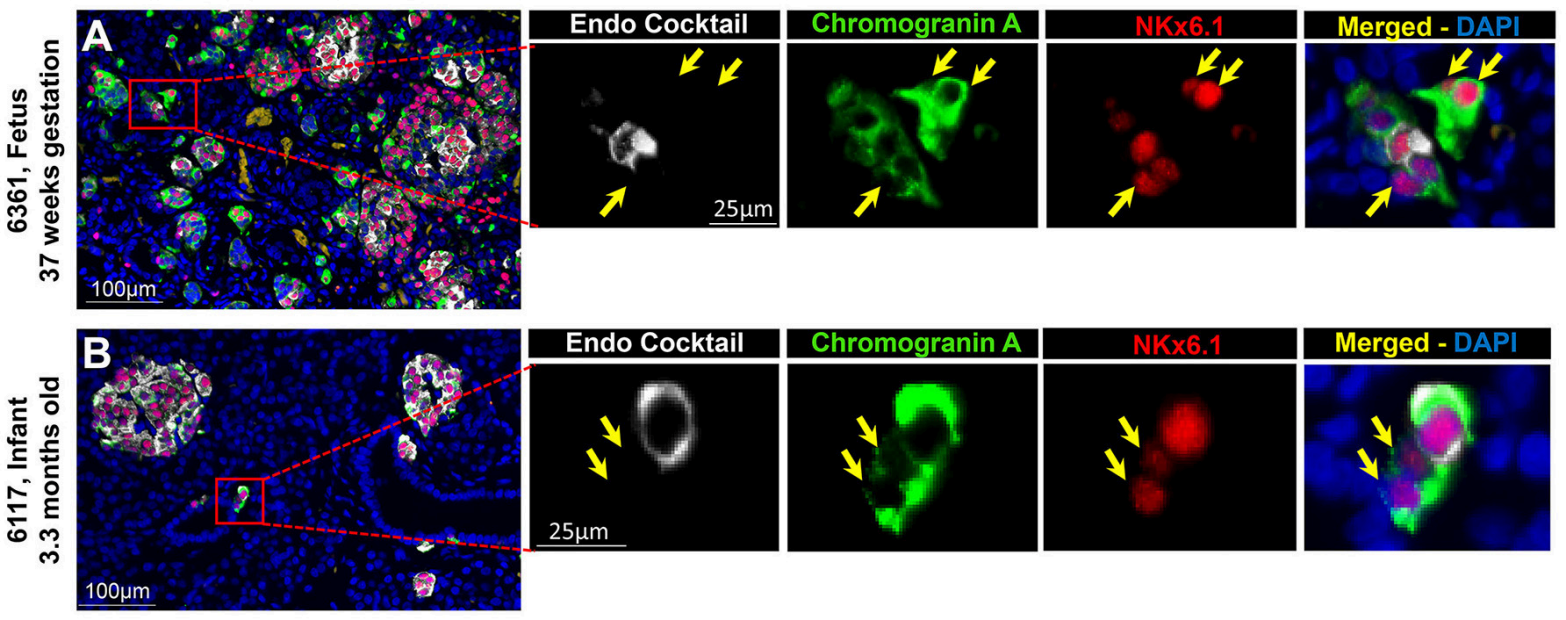
ChromograninA positive hormone-negative (CPHN) cells express the endocrine differentiation transcription factor NKX6.1 in both fetal and infant pancreas. Representative pancreatic sections from fetal **(A)** and infant **(B)** donors immunostained for Endocrine cocktail (insulin, glucagon, somatostatin, pancreatic polypeptide, and ghrelin) (white), chromograninA (green), the transcription factor NKX6.1 (red) and DAPI (blue). Yellow arrows indicate CPHN cells. Scale bars: 100 μm for low power and 25 μm for high magnification images.

**Figure 4:**
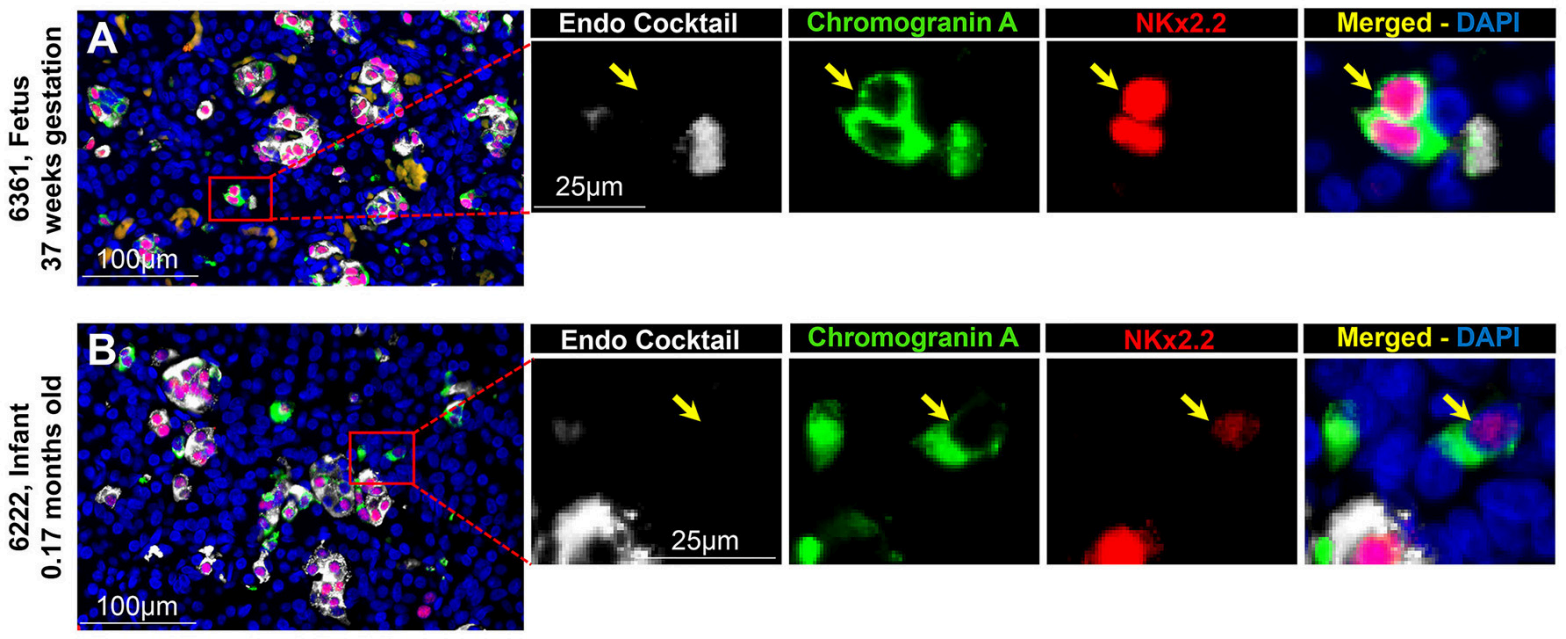
ChromograninA positive hormone-negative (CPHN) cells express the beta-cell differentiation transcription factor NKX2.2 in both fetuses and infants. Representative pancreatic sections from fetal **(A)** and infant **(B)** donors immunostained for Endocrine cocktail (insulin, glucagon, somatostatin, pancreatic polypeptide, and ghrelin) (white), chromograninA (green), the transcription factor NKX2.2 (red) and DAPI (blue). Yellow arrows indicate CPHN cells. Scale bars: 100 μm for low power and 25 μm for high magnification images.

**Figure 5:**
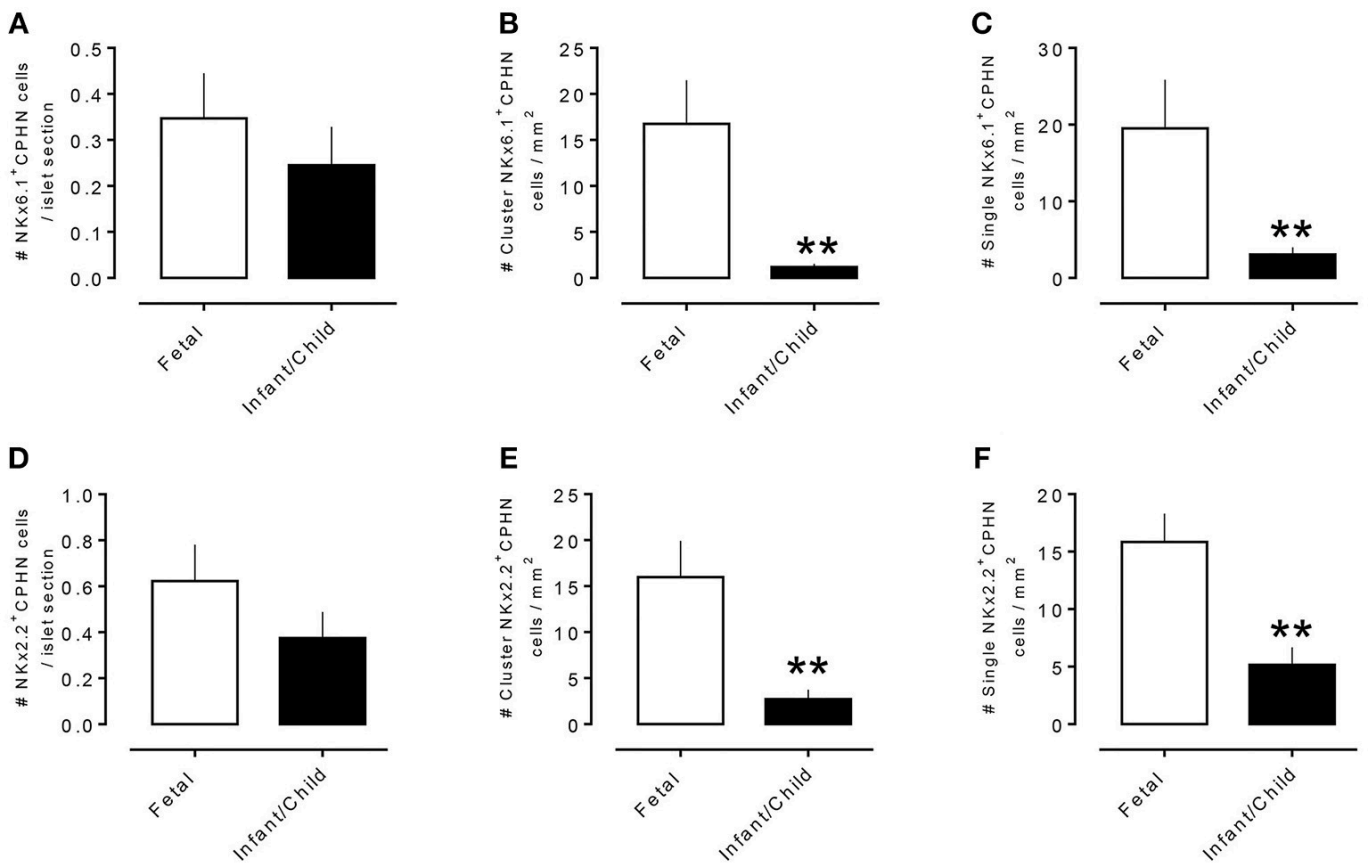
Comparison of number of CPHN cells expressing transcription factors NKX6.1 and NKX2.2 in different compartments of fetal and infant/child pancreas. CPHN cells that express either NKX6.1 or NKX2.2 showed variability in their numbers among different compartments of the pancreas sections of both fetal and infant cases. The number of CPHN cells located within the islets that expressed either NKX6.1 **(A)** or NKX2.2 **(D)** was similar in fetal and infant/child cases but NKX6.1^+^ and NKX2.2^+^ CPHN cells found as clusters **(B,E)** or single cells **(C,F)** were significantly higher in fetal cases compared to infant/child cases. ^**^p < 0.01, *n* = 7 for fetal cases and *n* = 10 for infant/child cases.

To evaluate the projected endocrine fate of CPHN cells, pancreatic tissue sections were stained with antibodies against the two major transcription factors involved in the specification and differentiation of the islet endocrine cells, NKX2.2 and NKX6.1. NKX2.2 or NKX6.1 positive CPHN cells were present in both fetal and infant/child pancreatic tissue (Figures 3A,B, 4A,B). Both NKX6.1^+^ and NKX2.2^+^ CPHN cells showed variability in their numbers among different compartments of the pancreas in both fetuses and infants [Supplementary Table 4, (19)]. The number of CPHN cells located within islets that expressed either NKX6.1 or NKX2.2 was similar in fetal and infant/child cases (0.34 ± 0.1 vs. 0.24 ± 0.08 NKX6.1^+^-CPHN cells/islet section, fetal vs. infant/child, *p* = ns and 0.62 ± 0.1 vs. 0.37 ± 0.1 NKX2.2^+^- CPHN cells/islet section, fetal vs. infant/child, *p* = ns) (Figures 5A,D). NKX6.1^+^ and NKX2.2^+^ CPHN cells were also found in greater abundance as clustered or single cells compared to cells within the islets and their frequency followed the pattern of being more abundant in fetal pancreas and decreasing with growth (16.7 ± 4.7 vs. 1.2 ± 0.3 clustered NKX6.1^+^-CPHN cells/mm^2^, fetal vs. infant/child, *p* < 0.01; 16.0 ± 4.0 vs. 2.7 ± 1.0 clustered NKX2.2^+^-CPHN cells/mm^2^, fetal vs. infant/child, *p* < 0.01) (Figures 5B,E). Likewise, the frequency of single NKX6.1^+^ and NKX2.2^+^ CPHN cells was also found to be higher in fetal cases compared to infant/child cases single (19.5 ± 6.3 vs. 3.0 ± 0.9 single NKX6.1^+^-CPHN cells/mm^2^ fetal vs. infant/child, *p* < 0.01; 15.8 ± 2.4 vs. 5.1 ± 1.5, single NKX2.2^+^-CPHN cells/mm^2^, *p* < 0.01) (Figures 5C,F). Since the size of the pancreas changes with age we also measured the frequency of NKX6.1 or NKX2.2 positive CPHN cells (in percentage) in fetal and infant subjects. The percentage of either NKX6.1^+^ or NKX2.2^+^ CPHN cells (of total CPHN cells) overall in all compartments was similar both in fetal and infant/child cases (81.9 ± 6.2 vs. 82.8 ± 3.8, % of NKX6.1^+^ CPHN cells overall in all compartments, fetal vs. infant/child, *p* = ns and 87.9 ± 4.7, vs. 82.1 ± 5.3, % of NKX2.2^+^ CPHN cells of total CPHN cells overall in all compartments, fetal vs. infant/child, *p* = ns) (Supplementary Figures 9A,E, (19)). Additionally, within islets the proportion was comparable both in fetal and infant/child cases (71.1 ± 12.0 vs. 81.3 ± 6.1% of NKX6.1^+^ CPHN cells in islets, fetal vs. infant/child, *p* = ns and 94.4 ± 4.6 vs. 76.0 ± 7.1% of NKX2.2^+^ CPHN cells in islets, *p* = ns) [Supplementary Figures 9B,F, (19)]. Likewise, the proportion of islet endocrine transcription factor positive clusters or single CPHN cells were also comparable between fetal and infant/child cases (83.7 ± 5.7 vs. 67.1 ± 12.0% of cluster NKX6.1^+^ CPHN cells, fetal vs. infant/child, *p* = ns and 94.4 ± 4.6 vs. 76.0 ± 7.1% of cluster NKX2.2^+^ CPHN cells; 89.5 ± 5.0 vs. 89.5 ± 5.0% single NKX6.1^+^ CPHN cells, fetal vs. infant/child and 89.1 ± 4.9 vs. 82.0 ± 10.3% single NKX2.2^+^ CPHN cells, fetal vs. infant/child, *p* = ns) [Supplementary Figures 9C, D,G,H, (19)].

## Discussion

During human fetal pancreas development, insulin expression is first detected at 8–9 weeks gestation (1, 10) and fractional beta cell area increases linearly until birth (1). Our data show that the frequency of CPHN cells declines during fetal to postnatal transition (4), possibly supporting the concept that these cells are programmed to mature into functional endocrine cells. A similar synaptophysin-positive, hormone-negative cell type has been reported in fetal human pancreas (9). These studies suggest the existence of hormone-negative precursor cell type prevalent in fetal human pancreas.

Islet formation in human pancreatic development involves a multistep rearrangement of endocrine cells, such that endocrine cells appear in the form of islets, as well as groups of small, scattered clusters in the exocrine tissue (1, 10, 23). Overall, ~12–13% of fetal endocrine cells are CPHN cells, distributed in islets and small endocrine cell clusters. Within islets, ~9% of the endocrine cells are CPHN cells, while a much higher proportion (~35%) of the scattered endocrine cells are CPHN cells at this stage of development. These small foci of endocrine cells are reminiscent of the pancreatic phenotype in pregnant humans where the increased beta-cell mass is coincident with an increase in scattered foci of beta-cells not derived by replication (24). Taken together, this supports the hypothesis that CPHN cells could possibly be precursors to endocrine cells.

Given the high rates of endocrine cell replication in late fetal pancreas development, we examined whether, as a potential precursor pool, CPHN cells would display a high replicative index. Our data show that CPHN cells replicate very infrequently compared to differentiated endocrine cells as well as the potential precursor PDG compartment. The low replication frequency of CPHN cells is comparable to the Ngn3-positive endocrine progenitors, which typically have a very low replication index (25, 26). If they do indeed represent a partially differentiated precursor population, the CPHN cells may not inherently be replication competent, perhaps to protect such a population from replicative stress. The susceptibility of partially differentiated endocrine cells to replicative stress has been widely documented, e.g., in the context of diabetes pathogenesis (27). Therefore, alternatively, these cells could represent a partially differentiated, misguided population that may fail to develop a mature endocrine identity and be fated for elimination via programmed cell death. One limitation of our study is that our fetal pancreatic samples are aged 19 weeks gestation onwards, such that the first time-point at which these cells emerge in the endocrine differentiation program is unclear.

NKX2.2 and NKX6.1 are key transcription factors that direct beta-cell development. NKX2.2 is required for endocrine cell specification and differentiation, and is present in beta-, as well as certain alpha-, and PP- cells in adults (28), while NKX6.1 is essential for beta-cell formation during embryonic development (29). The majority of CPHN cells in fetal and infant life are positive for NKX2.2 and NKX6.1, suggesting that most of these cells could possibly represent endocrine precursor cells and, if so, may differentiate into beta-cells. NKX6.1 lies downstream of NKX2.2 in the major pathway of beta cell formation (29). Moreover, while NKX2.2 appears in human embryos only after endocrine differentiation, both NKX6.1 and NKX2.2 are expressed in human fetal beta cells (30). Therefore, the high frequency of clustered or single NKX6.1 and NKX2.2 positive CPHN cells in human fetal pancreas suggests that the majority of CPHN cells may be programmed for a beta-cell lineage. In adults, it is equally possible that these cells underwent the maturation process and then lost some of their maturation signature through a dedifferentiation process, similar to what has been observed in stressed β cells (31, 32). However, the high frequency of clustered CPHN cells positive for NKX6.1 or NKX2.2 in fetal and infant cases in association with their limited replication capacity suggests that the CPHN cells might represent a partially differentiated cell type with distinct features of endocrine lineage rather than de-differentiated cells.

## Author Contributions

ASMM, CM, KZ, MN, SK, and AEB performed the studies, undertook the microscopy with assistance from MC, ASMM, and SD, and performed the morphological analysis. AEB, SD, HF, JM, RR, and MA researched data, wrote, reviewed and edited the manuscript, and contributed to the discussion.

### Conflict of Interest Statement

The authors declare that the research was conducted in the absence of any commercial or financial relationships that could be construed as a potential conflict of interest.

## Supplementary Material

Supplementary Figure 1 Human pancreatic development 1. A low power view of fetal pancreas **(A)** and infant pancreas **(B)** stained by immunohistochemistry for Insulin (pink), Glucagon (blue), and Ki67 (brown) with a hematoxylin counterstain. Insets, high power images of the indicated area marked by black squares in the low power images. Scale bars, 200 μm in low power images and 100 μm in insets.

Supplementary Figure 2 Human pancreatic development 2. A low power view of pancreas from young children, 2 years old **(A)** and 3.6 years old **(B)**, stained by immunohistochemistry for Insulin (pink), Glucagon (blue) and Ki67 (brown) with a hematoxylin counterstain. Insets, high power images of the indicated area marked by black squares in the low power images. Scale bars, 200 μm in low power images and 100 μm in insets.

Supplementary Figure 3 Human pancreatic development 3. A low power view of child pancreas, 6 years old **(A)** and 10 years old **(B)**, stained by immunohistochemistry for Insulin (pink), Glucagon (blue), and Ki67 (brown) with a hematoxylin counterstain. Insets, high power images of the indicated area marked by black squares in the low power images. Scale bars, 200 μm in low power images and 100 μm in insets.

Supplementary Figure 4 A rare example of replicating chromograninA positive hormone-negative (CPHN) cells in a fetal and an infant donor. Pancreatic sections from a fetal **(A)** and an infant **(B)** donor immunostained for Endocrine cocktail (insulin, glucagon, somatostatin, pancreatic polypeptide, and ghrelin) (white), chromograninA (green), Ki67 (red), and DAPI (blue). Yellow arrows showing Ki67 positive CPHN cells in one fetal and one infant donor, emphasizing that replication is a rare event in these cells. Scale bars: 100 μm for low power and 25 μm for high magnification images.

Supplementary Figure 5 Replication and expression of pan-endocrine hormones in cells in the ducts and PDGs of fetal and infant pancreas. Representative pancreatic sections from fetal and infant donors stained for Ki67/Hematoxylin (**A,B**, respectively) and Insulin/PP/hematoxylin (**C,D**, respectively). Insets, higher magnification of selected areas (indicated by black squares) in the low power images. Brown arrows (in **A,B** and their insets) indicate Ki67 staining (replication of cells) in ducts and PDGs. Brown arrows (insets of **C,D**) indicate expression of pancreatic polypeptide (PP) and purple arrows indicate expression of insulin in PDGs. Scale bars, 100 μm (for **A,B**), 200 μm (for **C,D**), 25 μm (for all the insets).

Supplementary Figure 6 Chromogranin A positive hormone-negative (CPHN) cells located in the pancreatic ducts do not replicate during fetal and infant life. Pancreatic ducts shown in tissue sections from fetal **(A)** and infant **(B)** donors immunostained for Endocrine cocktail (insulin, glucagon, somatostatin, pancreatic polypeptide and ghrelin) (white), chromograninA (green), Ki67 (red), and DAPI (blue). Yellow arrows indicate CPHN cells. Scale bars: 100 μm for low power and 25 μm for high magnification images.

Supplementary Figure 7 Replication of endocrine cells. Quantification of endocrine cell replication shown as percentage of Ki67 positive endocrine cells, immunostained with endocrine cocktail antibodies. Endocrine cell replication diminishes in the pancreas with age (*p* < 0.05).

Supplementary Figure 8 Examples of replicating islet endocrine cells in a fetal and an infant donor. Pancreatic sections from a fetal **(A)** and an infant **(B)** donor immunostained for Endocrine cocktail (insulin, glucagon, somatostatin, pancreatic polypeptide and ghrelin) (white), chromograninA (green), Ki67 (red), and DAPI (blue). Yellow arrows showing Ki67 positive endocrine cells in high power images indicated by red squares in low power images. The percentage of replication of islet endocrine cells decreased from fetal to postnatal life **(C)**. Scale bars: 50 μm for low power and 10 μm for high magnification images.

Supplementary Figure 9 Percent changes of CPHN cells (positive for either NKx6.1 or NKx2.2) in different compartments of fetal and infant/child pancreas with age: The percentage of either NKX6.1^+^ or NKX2.2^+^ CPHN cells (of total CPHN cells in fetal and infant/child cases) found in overall compartments **(A,E)**, within islets **(B,F)**, in cluster cells **(C,G)** or in single cells **(D,H)**.

Supplementary Table 1 Clinical characteristic of fetal and infant cases used for quantification of CPHN cells. PT, pancreas tail.

Supplementary Table 2 Clinical characteristics of nPOD fetal and infant donors for Ki67, Nkx2.2 and Nkx6.1 analysis. PH, pancreas head; PB, pancreas body; PT, pancreas tail.

Supplementary Table 3 Clinical characteristics of nPOD fetal and infant cases for Ki67 and hormone expression analysis in pancreatic ducts. PH, pancreas head; PB, pancreas body; PT, pancreas tail.

Supplementary Table 4 NKX6.1 + and NKX2.2 + CPHN cells detected in differentcompartments of the pancreas in fetal and infant donors.

## Abbreviations

CPHN: chromograninA positive hormone negative
NKX: homeobox protein family
PDX: pancreatic and duodenal homeobox
PDG: pancreatic duct glands.
