# Supplementary figures and images for: Characterization of Non-hormone Expressing Endocrine Cells in Fetal and Infant Human Pancreas
Source: Front Endocrinol (Lausanne). 2019 Jan 9;9:791. doi: 10.3389/fendo.2018.00791 (PMC6334491; doi:10.3389/fendo.2018.00791)

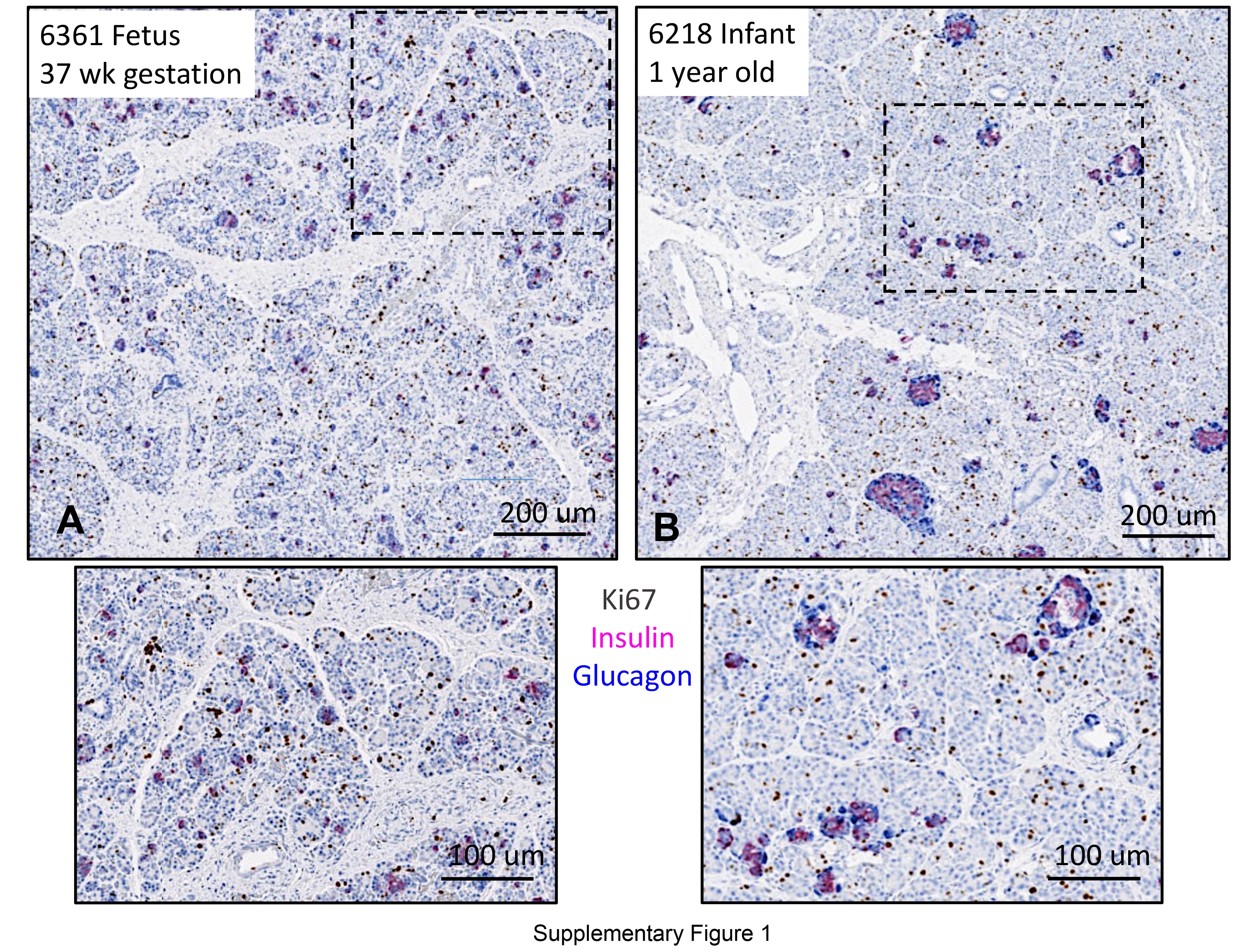

Supplement: Supplementary Figure 1 — Human pancreatic development 1. A low power view of fetal pancreas (A) and infant pancreas (B) stained by immunohistochemistry for Insulin (pink), Glucagon (blue), and Ki67 (brown) with a hematoxylin counterstain. Insets, high power images of the indicated area marked by black squares in the low power images. Scale bars, 200 μm in low power images and 100 μm in insets. [file Image_1.jpg]

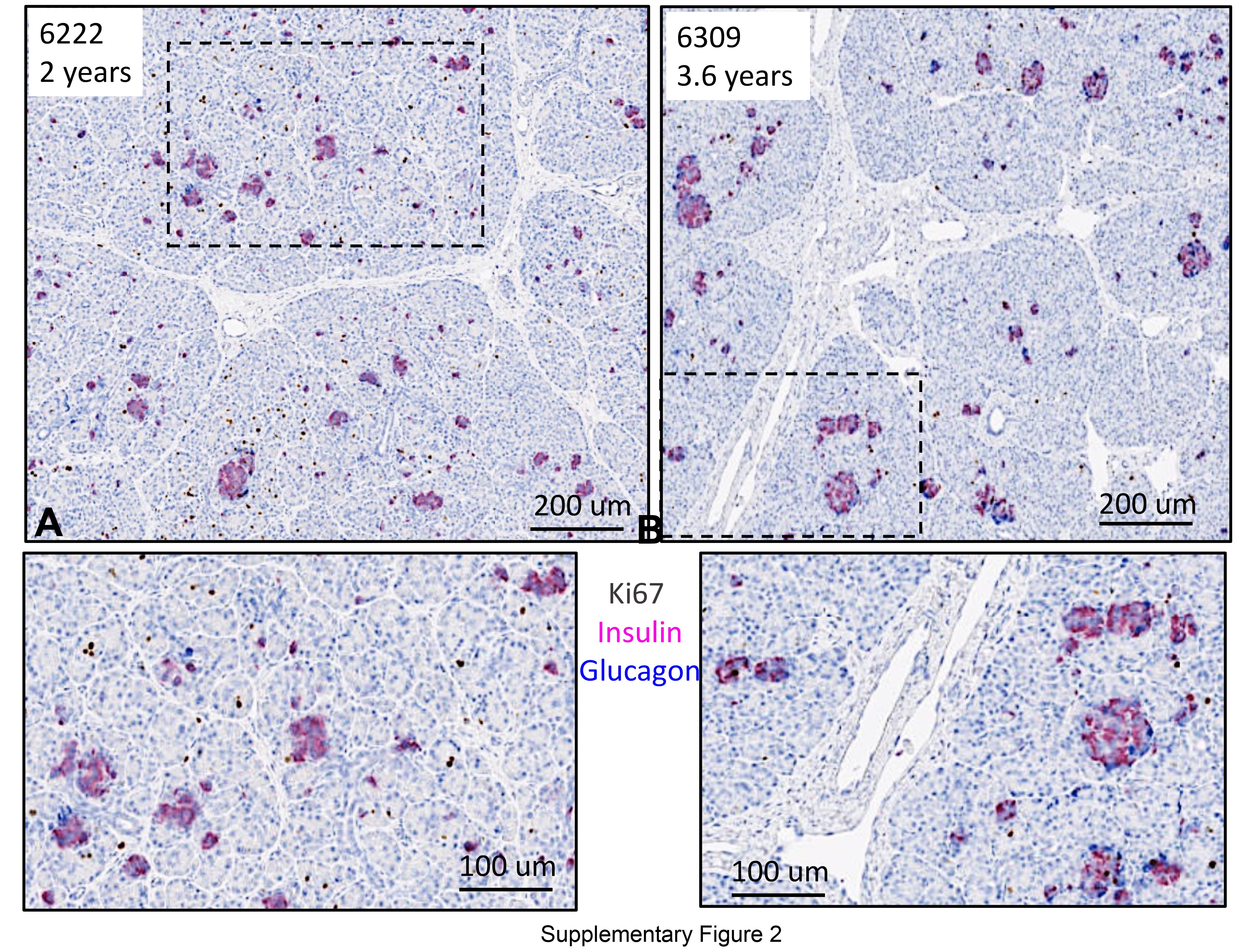

Supplement: Supplementary Figure 2 — Human pancreatic development 2. A low power view of pancreas from young children, 2 years old (A) and 3.6 years old (B), stained by immunohistochemistry for Insulin (pink), Glucagon (blue) and Ki67 (brown) with a hematoxylin counterstain. Insets, high power images of the indicated area marked by black squares in the low power images. Scale bars, 200 μm in low power images and 100 μm in insets. [file Image_2.jpg]

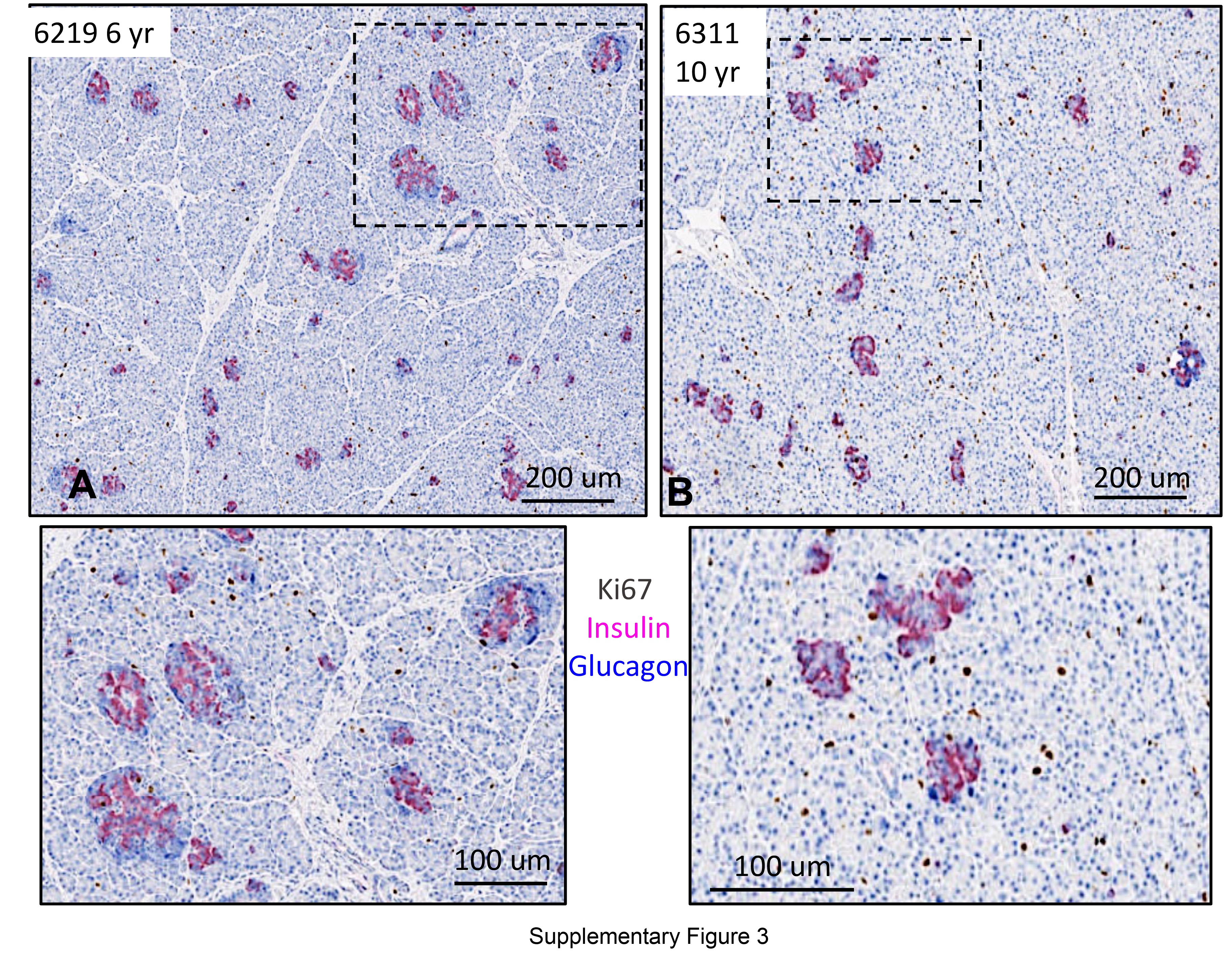

Supplement: Supplementary Figure 3 — Human pancreatic development 3. A low power view of child pancreas, 6 years old (A) and 10 years old (B), stained by immunohistochemistry for Insulin (pink), Glucagon (blue), and Ki67 (brown) with a hematoxylin counterstain. Insets, high power images of the indicated area marked by black squares in the low power images. Scale bars, 200 μm in low power images and 100 μm in insets. [file Image_3.jpg]

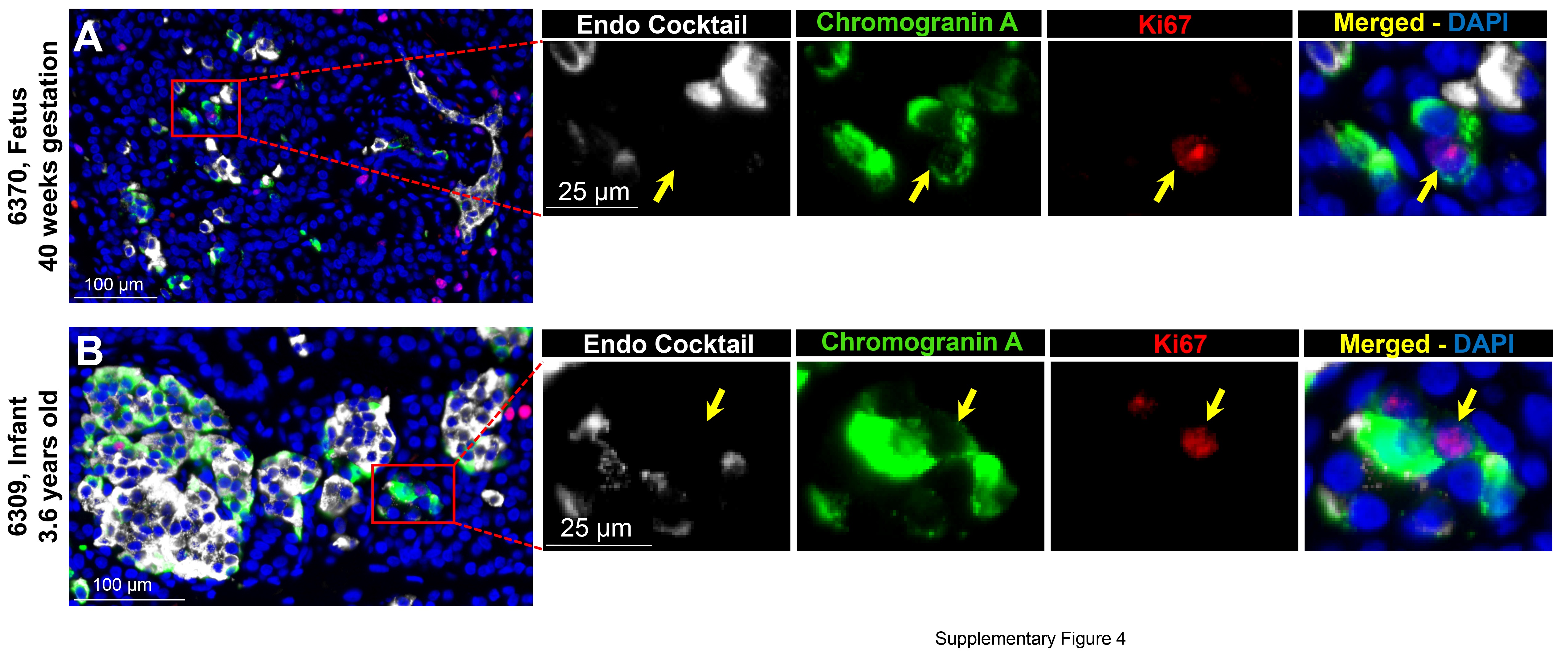

Supplement: Supplementary Figure 4 — A rare example of replicating chromograninA positive hormone-negative (CPHN) cells in a fetal and an infant donor. Pancreatic sections from a fetal (A) and an infant (B) donor immunostained for Endocrine cocktail (insulin, glucagon, somatostatin, pancreatic polypeptide, and ghrelin) (white), chromograninA (green), Ki67 (red), and DAPI (blue). Yellow arrows showing Ki67 positive CPHN cells in one fetal and one infant donor, emphasizing that replication is a rare event in these cells. Scale bars: 100 μm for low power and 25 μm for high magnification images. [file Image_4.jpg]

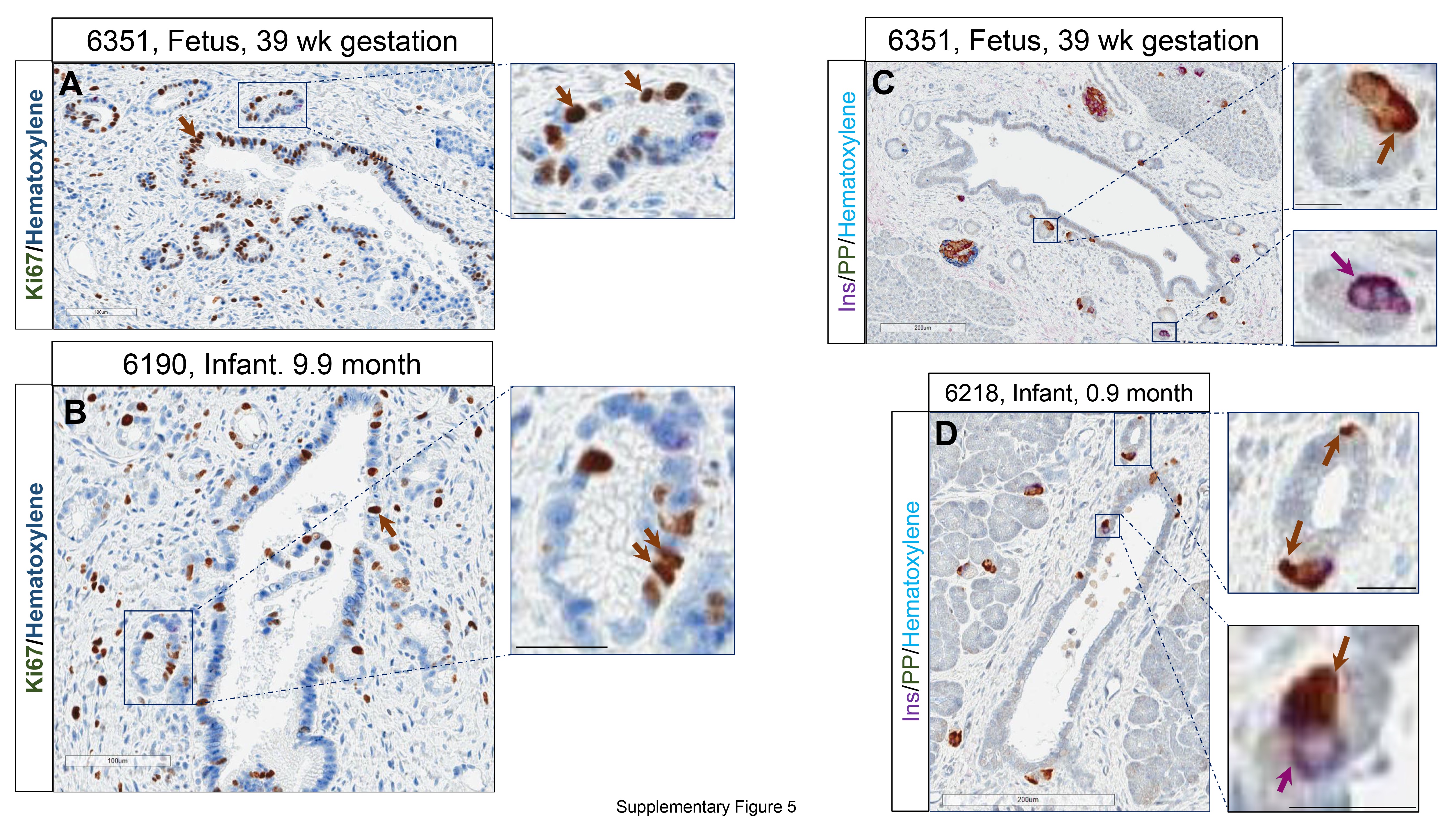

Supplement: Supplementary Figure 5 — Replication and expression of pan-endocrine hormones in cells in the ducts and PDGs of fetal and infant pancreas. Representative pancreatic sections from fetal and infant donors stained for Ki67/Hematoxylin (A,B, respectively) and Insulin/PP/hematoxylin (C,D, respectively). Insets, higher magnification of selected areas (indicated by black squares) in the low power images. Brown arrows (in A,B and their insets) indicate Ki67 staining (replication of cells) in ducts and PDGs. Brown arrows (insets of C,D) indicate expression of pancreatic polypeptide (PP) and purple arrows indicate expression of insulin in PDGs. Scale bars, 100 μm (for A,B), 200 μm (for C,D), 25 μm (for all the insets). [file Image_5.jpg]

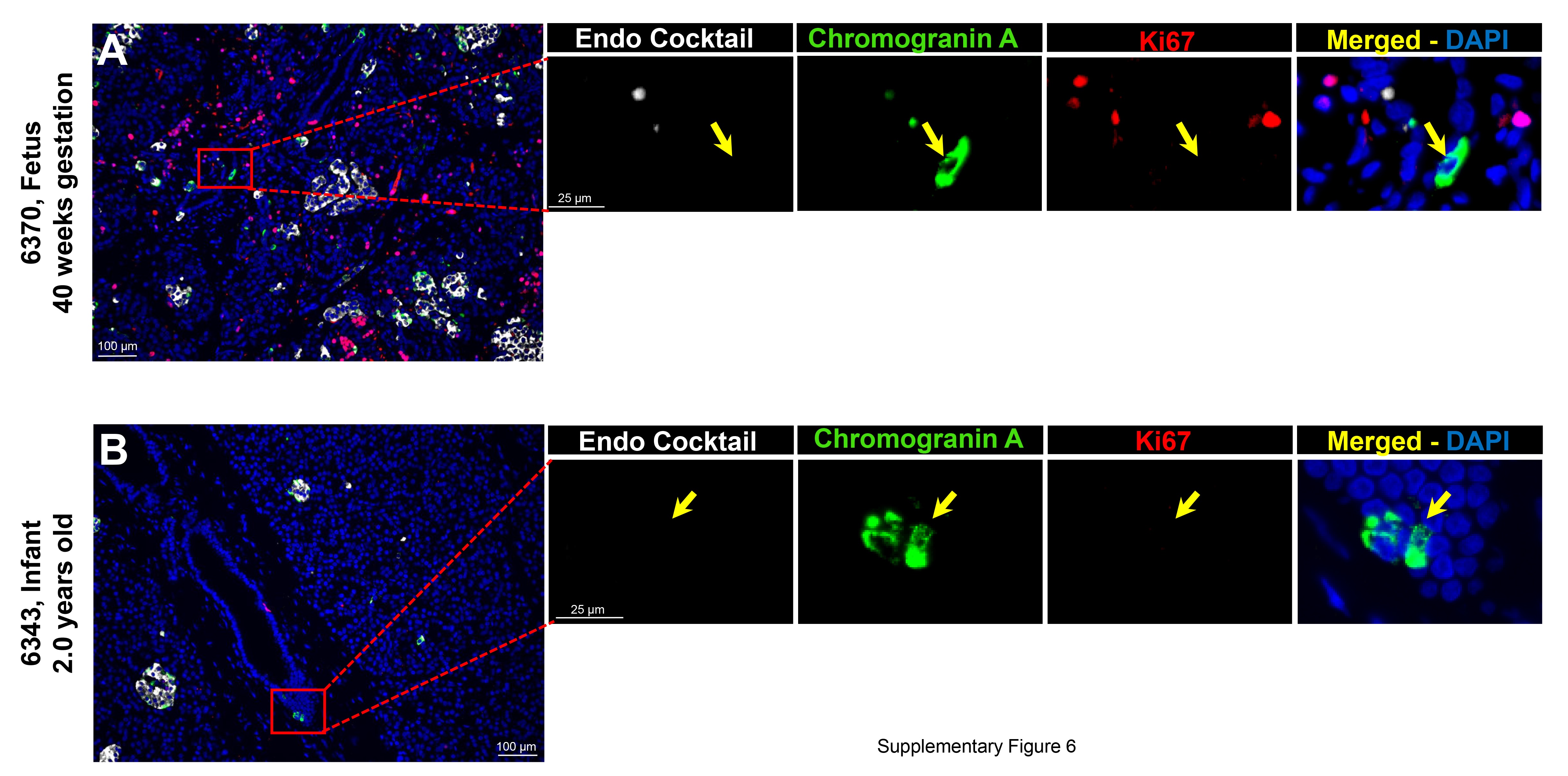

Supplement: Supplementary Figure 6 — Chromogranin A positive hormone-negative (CPHN) cells located in the pancreatic ducts do not replicate during fetal and infant life. Pancreatic ducts shown in tissue sections from fetal (A) and infant (B) donors immunostained for Endocrine cocktail (insulin, glucagon, somatostatin, pancreatic polypeptide and ghrelin) (white), chromograninA (green), Ki67 (red), and DAPI (blue). Yellow arrows indicate CPHN cells. Scale bars: 100 μm for low power and 25 μm for high magnification images. [file Image_6.jpg]

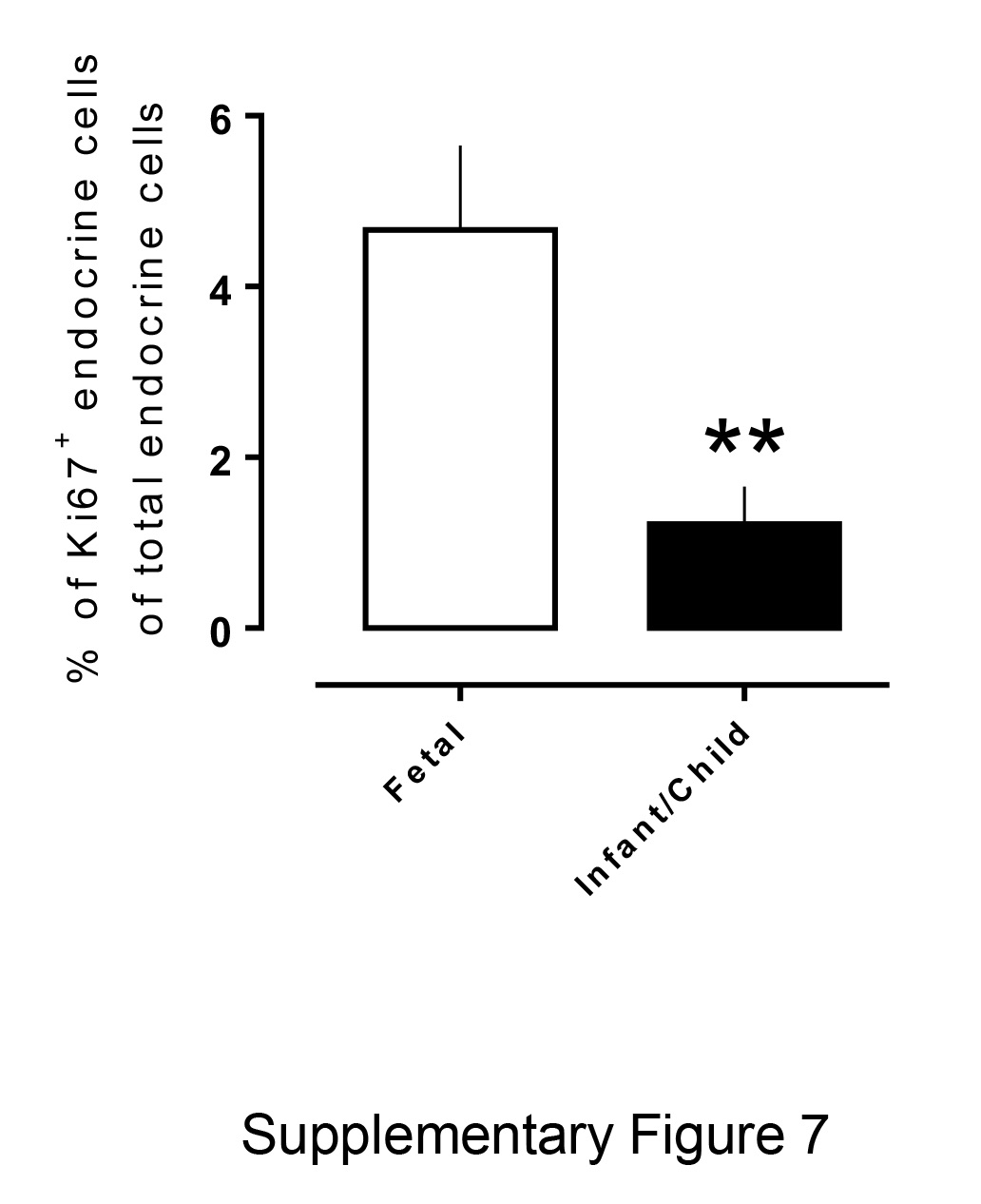

Supplement: Supplementary Figure 7 — Replication of endocrine cells. Quantification of endocrine cell replication shown as percentage of Ki67 positive endocrine cells, immunostained with endocrine cocktail antibodies. Endocrine cell replication diminishes in the pancreas with age (p < 0.05). [file Image_7.jpg]

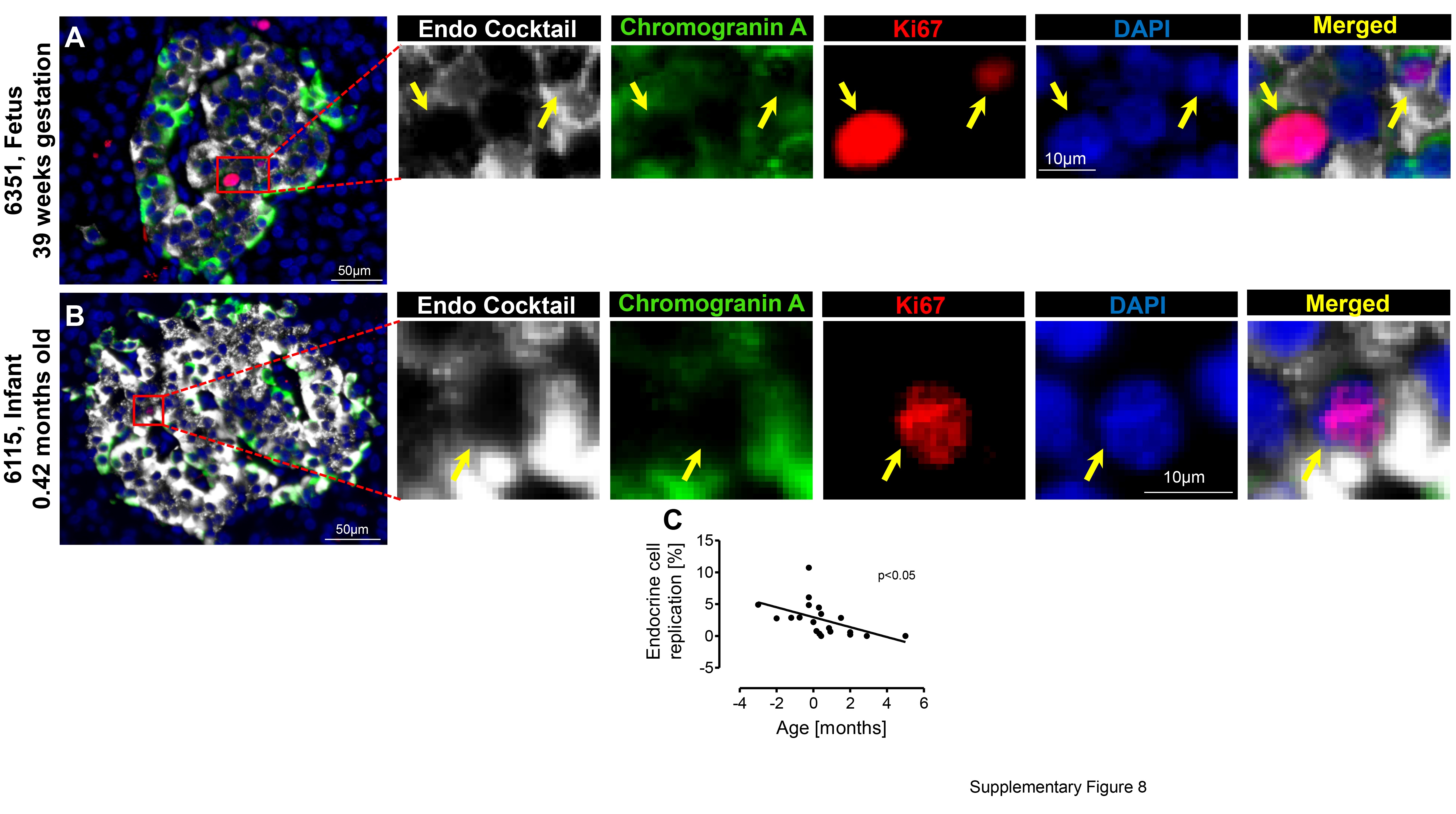

Supplement: Supplementary Figure 8 — Examples of replicating islet endocrine cells in a fetal and an infant donor. Pancreatic sections from a fetal (A) and an infant (B) donor immunostained for Endocrine cocktail (insulin, glucagon, somatostatin, pancreatic polypeptide and ghrelin) (white), chromograninA (green), Ki67 (red), and DAPI (blue). Yellow arrows showing Ki67 positive endocrine cells in high power images indicated by red squares in low power images. The percentage of replication of islet endocrine cells decreased from fetal to postnatal life (C). Scale bars: 50 μm for low power and 10 μm for high magnification images. [file Image_8.jpg]

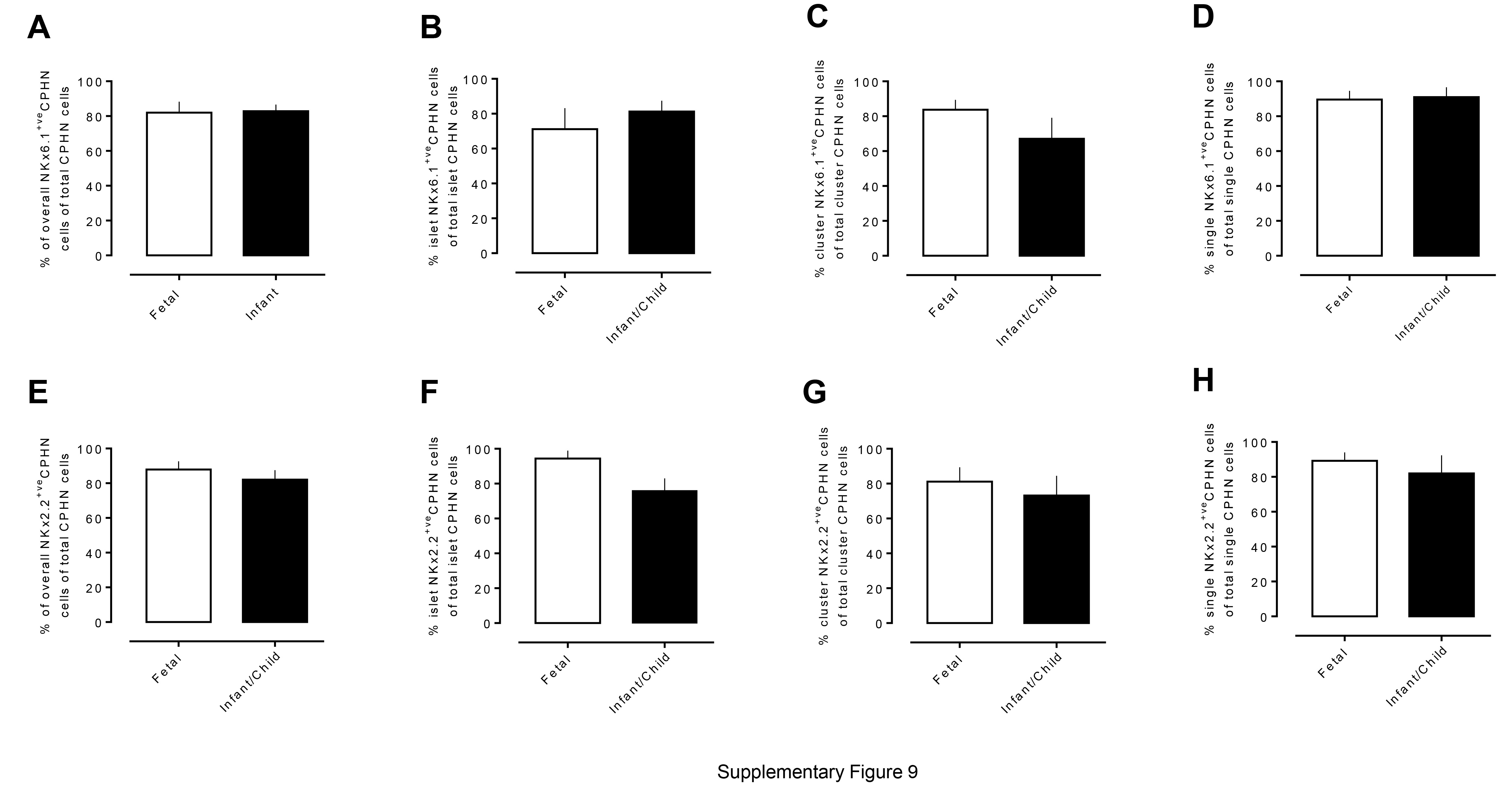

Supplement: Supplementary Figure 9 — Percent changes of CPHN cells (positive for either NKx6.1 or NKx2.2) in different compartments of fetal and infant/child pancreas with age: The percentage of either NKX6.1+ or NKX2.2+ CPHN cells (of total CPHN cells in fetal and infant/child cases) found in overall compartments (A,E), within islets (B,F), in cluster cells (C,G) or in single cells (D,H). [file Image_9.jpg]
