# Supplementary material for: Characterization of Non-hormone Expressing Endocrine Cells in Fetal and Infant Human Pancreas
Source: Front Endocrinol (Lausanne). 2019 Jan 9;9:791. doi: 10.3389/fendo.2018.00791 (PMC6334491; doi:10.3389/fendo.2018.00791)
Supplement: Supplementary Table 1 — Clinical characteristic of fetal and infant cases used for quantification of CPHN cells. PT, pancreas tail. [file Table_1.DOCX]

**Supplementary Table 1. Clinical characteristic of fetal and infant cases used for quantification of CPHN cells.**

| **ID/ Portion of pancreas studied** | **Age** | **Sex** | **Cause of Death** |
| --- | --- | --- | --- |
| **Fetal** | weeks |  | |
| **1/PT** | 26 | F | X |
| **2/PT** | 30 | M | X |
| **3/PT** | 32 | F | X |
| **4/PT** | 34 | M | X |
| **5/PT** | 25 | F | X |
| **6/PT** | 20 | F | X |
| **7/PT** | 19 | F | X |
| **8/PT** | 20 | F | X |
| **Infant/child** | months |  | |
| **1/PT** | 0.01 | M | Respiratory failure |
| **2/PT** | 0.07 | M | Congenital heart disease |
| **3/PT** | 0.7 | F | Congenital heart disease |
| **4/PT** | 0.7 | M | Congenital heart disease |
| **5/PT** | 1.25 | M | Congenital heart disease |
| **6/PT** | 1.5 | F | Septal defect |
| **7/PT** | 2.25 | F | Congenital heart disease |
| **8/PT** | 3 | M | Congenital heart disease |
| **9/PT** | 3 | F | Sudden infant death |
| **10/PT** | 3 | F | Sudden infant death |
| **12/PT** | 7 | F | Hypoxic encephalopathy |
| **13/PT** | 8 | F | Respiratory failure |
| **14/PT** | 10 | F | Head injury due to motor vehicle accident |
| **15/PT** | 11 | F | Biliary atresia |
| **16/PT** | 12.5 | F | Congenital heart disease |
| **17/PT** | 13 | F | Cardiac failure |
| **18/PT** | 14 | M | Congenital heart disease |
| **19/PT** | 23 | M | Hypoxic encephalopathy |

**PT, pancreas tail.**
