# Supplementary material for: Characterization of Non-hormone Expressing Endocrine Cells in Fetal and Infant Human Pancreas
Source: Front Endocrinol (Lausanne). 2019 Jan 9;9:791. doi: 10.3389/fendo.2018.00791 (PMC6334491; doi:10.3389/fendo.2018.00791)
Supplement: Supplementary Table 2 — Clinical characteristics of nPOD fetal and infant donors for Ki67, Nkx2.2 and Nkx6.1 analysis. PH, pancreas head; PB, pancreas body; PT, pancreas tail. [file Table_2.DOCX]

**Supplementary Table 2. Clinical characteristics of nPOD fetal and infant donors for Ki67, Nkx2.2 and Nkx6.1 analysis.**

| **ID/Portion of pancreas studied** | **Age** | **Sex** | **Cause of Death** | **Used for Ki67 analysis** | **Used for Nkx6.1 or NKx2.2 analysis** |
| --- | --- | --- | --- | --- | --- |
| **Fetal** | **weeks** |  |  |  |  |
| **6192/PB** | 28 | F | Hydrops fetalis | X |  |
| **6200/PH** | 32 | F | Pulmonary hypoplasia | X | X |
| **6214/PH** | 35.5 | M | Tetralogy of Fallot | X | X |
| **6361/PH** | 37 | M | X | X | X |
| **6348/PH** | 39 | F | Placental abruption | X | X |
| **6349/PH** | 39 | F | Anencephaly | X | X |
| **6351/PH** | 39 | M | Placental abruption | X | X |
| **6370/PH** | 40 | M | Anencephaly | X | X |
| **Infant/child** | **months** |  | |  |  |
| **6222/PH** | 2.04 | M | Sudden infant death | X | X |
| **6305/PB** | 3 | M | Sudden infant death | X |  |
| **6309/PB** | 3.6 | M | Intracranial hemorrhage | X |  |
| **6117/PB** | 3.96 | M | Motor vehicle accident | X | X |
| **6122/PB** | 5.04 | F | Subdural hemorrhage | X | X |
| **6115/PB** | 5.04 | M | Cardiopulmonary arrest | X | X |
| **6376/PH** | 7.2 | F | Sudden death, cause unknown | X |  |
| **6311/PH** | 10.2 | M | Subdural hemorrhage | X |  |
| **6408/PH** | 11.04 | F | X | X |  |
| **6103/PH** | 18 | M | Cardiopulmonary arrest | X | X |
| **6315/PB** | 19.2 | M | Subdural hemorrhage | X |  |
| **6343/PB** | 24 | M | Subdural hemorrhage | X | X |
| **6014/PT** | 24 | M | Drowning accident | X | X |
| **6094/PH** | 32.4 | M | Drowning accident | X | X |
| **6292/PH** | 36 | M | Intracerebral hemorrhage | X | X |
| **6005/PH** | 60 | F | Intracranial hemorrhage | X | X |

**PH, pancreas head; PB, pancreas body: PT, pancreas tail.**
