# Supplementary material for: Characterization of Non-hormone Expressing Endocrine Cells in Fetal and Infant Human Pancreas
Source: Front Endocrinol (Lausanne). 2019 Jan 9;9:791. doi: 10.3389/fendo.2018.00791 (PMC6334491; doi:10.3389/fendo.2018.00791)
Supplement: Supplementary Table 3 — Clinical characteristics of nPOD fetal and infant cases for Ki67 and hormone expression analysis in pancreatic ducts. PH, pancreas head; PB, pancreas body; PT, pancreas tail. [file Table_3.DOCX]

**Supplementary Table 3. Clinical characteristics of nPOD fetal and infant cases for Ki67 and hormone expression analysis in pancreatic ducts.**

| **ID/ Portion of pancreas studied** | **Age** | **Sex** | **Cause of Death** |
| --- | --- | --- | --- |
| **Fetal** | **weeks** |  | |
| **6200/PH** | 32 | F | Pulmonary hypoplasia |
| **6214/PH** | 35.5 | M | Tetralogy of Fallot |
| **6201/PH** | 36 | M | Anencephaly |
| **6361/PH** | 37 | M | X |
| **6348/PH** | 39 | F | Placental abruption |
| **6349/PH** | 39 | F | Anencephaly |
| **6351/PH** | 39 | M | Placental abruption |
| **6370/PH** | 40 | M | Anencephaly |
| **6346/PH** | 40 | F | Anencephaly |
| **Infant/child** | **months** |  | |
| **6218/PH** | 0.96 | F | Sudden infant death |
| **6222/PH** | 2.04 | M | Sudden infant death |
| **6305/PB** | 3 | M | Sudden infant death |
| **6183/PB** | 3.6 | M | Pulmonary hypertension |
| **6309/PB** | 3.6 | M | Intracranial hemorrhage |
| **6117/PB** | 3.96 | M | Motor vehicle accident |
| **6187PH** | 4.8 | M | Subdural hemorrhage |
| **6122/PB** | 5.04 | F | Subdural hemorrhage |
| **6125/PH** | 5.04 | M | Meningitis |
| **6115/PB** | 5.04 | M | Cardiopulmonary arrest |
| **6219/PB** | 6 | M | Cardiopulmonary arrest |
| **6376/PH** | 7.2 | F | Sudden death, unknown |
| **6190/PH** | 9.96 | M | Genetic defects, multiple |
| **6311/PH** | 10.2 | M | Subdural hemorrhage |
| **6408/PH** | 11.04 | F | X |
| **6103/PH** | 18 | M | Cardiopulmonary arrest |
| **6315/PB** | 19.2 | M | Subdural hemorrhage |
| **6343/PB** | 24 | M | Subdural hemorrhage |
| **6014/PT** | 24 | M | Drowning accident |
| **6182/PH** | 32.4 | M | Seizure |
| **6094//PH** | 34.8 | M | Drowning accident |
| **6292/PH** | 36 | M | Intracerebral hemorrhage |
| **6005/PH** | 60 | F | Intracranial hemorrhage |

**PH, pancreas head; PB, pancreas body; PT, pancreas tail**
