# Supplementary material for: Characterization of Non-hormone Expressing Endocrine Cells in Fetal and Infant Human Pancreas
Source: Front Endocrinol (Lausanne). 2019 Jan 9;9:791. doi: 10.3389/fendo.2018.00791 (PMC6334491; doi:10.3389/fendo.2018.00791)
Supplement: Supplementary Table 4 — NKX6.1 + and NKX2.2 + CPHN cells detected in differentcompartments of the pancreas in fetal and infant donors. [file Table_4.DOCX]

**Supplementary Table 4: NKX6.1 + and NKX2.2 + CPHN cells detected in different
compartments of the pancreas in fetal and infant donors**

|  | **% of NKX6.1^+^ CPHN cells of total CPHN cells** | | | | **% of NKX2.2^+^ CPHN cells of total CPHN cells** | | | |
| --- | --- | --- | --- | --- | --- | --- | --- | --- |
|  | In overall compartment | Within islets | In clusters | In single cells | In overall compartment | Within islets | In clusters | In single cells |
| Fetal | 82.0 ± 6.3 | 71.1 ± 12.1 | 83.7 ± 5.7 | 89.6 ± 5.0 | 87.9 ± 4.7 | 94.4 ± 4.6 | 81.2 ± 8.2 | 89.2 ± 4.9 |
| Infant | 82.8 ± 3.8 | 81.3 ± 6.1 | 67.1 ± 12.1 | 91.0 ± 5.6 | 82.1 ± 5.4 | 75.9 ± 7.1 | 73.2 ± 11.3 | 82.0 ± 10.3 |
|  |  |  |  |  |  |  |  |  |
|  | **Average number of NKX6.1^+^ CPHN cells** | | | | **Average number of NKX2.2^+^ CPHN cells** | | | |
|  | In overall compartment | Per islet section | In clusters / mm^2^ | In single cells / mm^2^ | In overall compartment | Per islet section | In clusters / mm^2^ | In single cells / mm^2^ |
| Fetal | 60.2 ± 15.1 | 0.3 ± 0.1 | 16.7 ± 4.7 | 19.5 ± 6.3 | 60.6 ± 12.6 | 0.6 ± 0.2 | 16.0 ± 4.0 | 15.8 ± 2.5 |
| Infant | 8.8 ± 1.9 | 0.2 ± 0.1 | 1.2 ± 0.3 | 3.1 ± 0.9 | 17.2 ± 4.2 | 0.4 ± 0.1 | 2,7 ± 1.0 | 5.1 ± 1.5 |
